# Genetic engineering of the precursor supply pathway for the overproduction of the *n*C_14_-surfactin isoform with promising MEOR applications

**DOI:** 10.1186/s12934-021-01585-4

**Published:** 2021-05-08

**Authors:** Fangxiang Hu, Weijie Cai, Junzhang Lin, Weidong Wang, Shuang Li

**Affiliations:** 1 College of Biotechnology and Pharmaceutical Engineering, Nanjing Tech University, Nanjing, 211816 PR China; 2 Oil Production Research Institute, Shengli Oil Field Ltd. Co. Sinopec, Dongying, 257000 PR China

**Keywords:** Lipopeptide, Surfactin, Thioesterase, *Bacillus subtilis*, Microbial Enhanced Oil Recovery (MEOR)

## Abstract

**Background:**

Surfactin, a representative biosurfactant of lipopeptide mainly produced by *Bacillus subtilis,* consists of a cyclic heptapeptide linked to a β-hydroxy fatty acid chain. The functional activity of surfactin is closely related to the length and isomerism of the fatty acid chain.

**Results:**

In this study, the fatty acid precursor supply pathway in *Bacillus subtilis* 168 for surfactin production was strengthened through two steps. Firstly, pathways competing for the precursors were eliminated with inactivation of *pps* and *pks*. Secondly, the plant medium-chain acyl-carrier protein (ACP) thioesterase (BTE) from *Umbellularia californica* was overexpressed. As a result, the surfactin titer after 24 h of cultivation improved by 34%, and the production rate increased from 0.112 to 0.177 g/L/h. The isoforms identified by RP-HPLC and GC–MS showed that the proportion of *n*C_14_-surfactin increased 6.4 times compared to the control strain. A comparison of further properties revealed that the product with more *n*C_14_-surfactin had higher surface activity and better performance in oil-washing. Finally, the product with more *n*C_14_-surfactin isoform had a higher hydrocarbon-emulsification index, and it increased the water-wettability of the oil-saturated silicate surface.

**Conclusion:**

The obtained results identified that enhancing the supply of fatty acid precursor is very essential for the synthesis of surfactin. At the same time, this study also proved that thioesterase BTE can promote the production of *n*C_14_-surfactin and experimentally demonstrated its higher surface activity and better performance in oil-washing. These results are of great significance for the MEOR application of surfactin.

**Graphic abstract:**

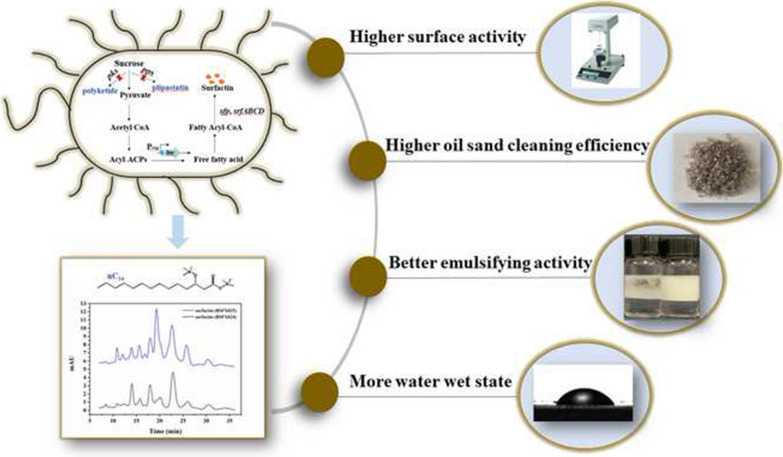

## Background

Microbial enhanced oil recovery (MEOR) has been widely used in the field of crude oil extraction as an environmentally-friendly and biodegradable alternative to chemical surfactants [1, 2]. Strategies for the application of MEOR include in-situ and ex-situ approaches. The former often relies on injecting nutrients to activate the microbes that generate bioproducts inside the reservoir [3], while the latter is based on direct injection of highly concentrated fermented functional products into oil reservoirs to improve oil recovery [4]. During the ex-situ MEOR process, functional products such as biosurfactants can be used directly as oil-displacing agents without considering the microorganism's adaptability to the extreme conditions inside the reservoir [5]. Among the reported biosurfactants, the cyclic lipopeptide surfactin has demonstrated the greatest potential for oilfield applications.

Surfactin is a secondary metabolite produced by *Bacillus* strains that can reduce the surface tension of water from 72 to 27 mN/m at a concentration of 1 × 10^–5^ mol/L (10 mg/L) [6]. Moreover, it also possesses advantages of high temperature stability up to 121 °C [7], high salt tolerance [8], wide pH adaptability [9] and high interfacial activity [10]. Based on these characteristics, a number of studies have shown that surfactin-based extraction has good prospects in MEOR [11]. The industrial application of surfactin is not limited by yield, since the surfactin production of engineered strains reached 10–20 g/L [12]. At present, the point of concern is the heterogeneity of surfactin structure, which closely affects the functional activity of surfactin preparations.

Surfactin is composed of a β-hydroxy fatty acid chain with a length of 13–15 carbon atoms connected to a peptide ring of 7 amino acids (L-Glu-L-Leu-D-Leu-L-Val-L-Asp-D-Leu-L-Leu) [13]. The isoforms of β-hydroxy fatty acid chain are usually branched, accounting for about 78% of the total. The main reason is that beta-ketoacyl-acyl carrier protein synthase III (FabH) involved in the initial steps of the straight- and branched-chain fatty acid synthesis cycle shows higher activity and selectivity for branched-chain fatty acid synthesis precursors [14–16]. The isoforms of the β-hydroxy fatty acid moiety mainly include *iso*, *anteiso* and straight (*n*) chains, mainly with chain lengths of C_14_ and C_15_ [17, 18]. Youssef et al. [14]analyzed the relationship between the surface activity and the fatty acyl structure of 8 surfactin isomers, and the results showed that the iso-odd fatty acyl isomer has higher oil displacement activity than the n-even fatty acyl isomers. Razafindralamb et al. [19] proved that C_14_ surfactin has higher foaming capacity than C_13_ and C_15_ surfactin. Our own research also showed that a higher C_15_-surfactin content results in better oil-washing and oil-displacement efficiency [11]. Therefore, the composition of surfactin isoforms in the reparation merits more attention than the total output of surfactin.

At present, strategies proposed to modify the structure of surfactin are all based on the metabolism of branched-chain amino acids, because some precursors for the synthesis of branched chain fatty acids presented in surfactin are derived from the branched chain amino acids valine, leucine and isoleucine [12]. For example, the proportion of surfactin with even β-hydroxy fatty acid components C_14_ and C_16_ increased with the addition of Arg, Gln or Val, whereas the addition of Cys, His, Ile, Leu, Met, and Ser enhanced the proportion of odd β-hydroxy fatty acids in *B. subtilis* TD7 [20]. The proportion of *n*C_14_ surfactin was increased 2.5 times by knocking out the *lpdV* gene, located in the *bkd* operon (*lpdV*, *bkdAA*, *bkdAB* and *bkdB* genes), which is responsible for the last step of the branched chain amino acid (BCAA) degradation in acyl-CoA [17].

Here, the importance of the fatty acid precursor supply for modifying the fatty acyl moiety of surfactin was proven again. The proportion of *n*C_14_-surfactin was increased after the elimination of pathways competing for the precursors and overexpression of thioesterase BTE. The biosurfactant production, surfactin isoforms composition, and enhanced oil recovery properties including surface activity, oil-washing efficiency, emulsifying activity and wettability alteration were analyzed, revealing promising application potential of the production strain developed in this study.

## Materials and methods

### Reagents and strain construction

Yeast extract and peptone were purchased from Oxoid (Hampshire, England). BSTFA (N,O-bis(trimethylsilyl)-trifluoroacetamide) was purchased from Macklin (Shanghai, China). Other chemicals were purchased from the China National Pharmaceutical Group Corporation (Shanghai, China).

All strains and plasmids used in this study are listed in Table 1. The marker-free knockout and knock-in approach has been described in detail before [21, 22]. Briefly, the left flanking region (LF) (∼ 800 bp), target genes (IG), direct repeat (DR) sequence (∼ 500 bp), PC cassette (1900 bp), and right flanking (RF) region (∼ 800 bp) fragments were first amplified using appropriate primers (Table 2), and then fused using overlap-extension PCR in the order LF, DR, PC cassette, and RF or LF, IG, DR, PC cassette, and RF. The resulting purified PCR products LF-DR-PC-RF/ LF-IG-DR-PC-RF were used to transform corresponding competent cells and further selected on chloramphenicol-containing agar plates and MGY-Cl medium. Oligonucleotide primers were synthesized by GenScript (Nanjing, China). The transformation of *Bacillus* was carried out according to published protocols [23, 24]. *B. subtilis* BSFX022 was constructed in previous research by overexpressing 4′ phosphopantetheinyl transferase, medium-chain acyl-acyl carrier protein (ACP) thioesterase and fatty acyl CoA ligase (encoded by *sfp*, *bte*, and y*hfL*, respectively), and knocking out acyl-CoA dehydrogenase (encoded by *fadE*) [20]. Here, BSFX022 was used as the parental strain to construct the strains BSFX023, BSFX024, BSFX025 and BSFX026. The strains BSFX023, BSFX024 and BSFX026 were constructed using the knockout method as reported before, while the recombinant strain BSFX025 was constructed using the knock-in method [21]. The nucleotide sequence of the promoter P_veg_ refers to reference [25] was synthesized by GENEWIZ. The P_veg_-*bte* cassette in strain BSFX025 was constructed through overlap PCR. Firstly, P_veg_ gene fragment was amplified by primer pairs P_veg_-F /P_veg_-R using the synthesized nucleotide sequence as a template. Gene fragment *bte* was amplified from plasmid pBTE using primer pairs bte-F /bte-R. Then, P_veg_ and *bte* were fused to generate P_veg_-*bte* using primer*s* P_veg_-F/ bte-R.

**Table 1:** Bacterial strains and plasmids used in this study

Strain or plasmid	Characteristics	Source
Plasmids		
pTPC	pMD19-T harboring the PC cassette	[22]
pBTE	pUC18 derivative containing cloned BTE	[21]
Strains		
BSFX022	*B. subtilis* 168 derivative, *sfp*^+^*yhfl*^+^*P*_*43*_*-bte*^+^*,* Δ*fadE*	[21]
BSFX023	BSFX022 derivative, Δ*pps*	This work
BSFX024	BSFX023 derivative, Δ*pks*	This work
BSFX025	BSFX024 derivative, insertion of *bte* under the control of the P_veg_ promoter, Δ*ackA*	This work
BSFX026	BSFX024 derivative, Δ*ackA*	This work
168 (P_veg_-GFP)	*B. subtilis 168* derivative, insertion of P_veg_-*gfp* cassette at the locus of *amyE*, Δ*amyE*	This work
168 (P_43_-GFP)	*B. subtilis 168* derivative, insertion of P_43_-*gfp* cassette at the locus of *amyE*, Δ*amyE*	This work

**Table 2:** Primers used in this study

Primer	Sequence
PC-F	ATTTTTAAAGTATGTATACAAATGA
PC-R	TTATAAAAGCCAGTCATTAGGCCTA
pps-LF-F	TTTATTTGAAAGGGAAAGGCGATCC
pps-LF-R	AATGGCCTCTGTCCGCTAATCCGCTCGGATTCCCTCCAGTTCTCATAATA
pps-DR-F	TATTATGAGAACTGGAGGGAATCCGAGCGGATTAGCGGACAGAGGCCT
pps-DR-R	TCATTTGTATACATACTTTAAAAATAATGGCCTCTGTCCGCTAATCCGCT
pps-RF-F	TAGGCCTAATGACTGGCTTTTATAATTGAGCGAACATACTTATTCTTTAA
pps-RF-R	CAAGGTGCGCAGCCAGCCGGCTGGC
pks-LF-F	AGCGTATGTGATGCCAAGTATGGAG
pks-LF-R	AGAATCGCTTTTCACACTAGTGCCTAGCTTTATTGTAACAAGAAAAAAT
pks-DR-F	ATTTTTTTCTTGTTACAATAAAGCTAGGCACTAGTGTGAAAAGCGATTCT
pks-DR-R	TCATTTGTATACATACTTTAAAAATAAAATACTCTCAGAAAACAAATAC
pks-RF-F	TAGGCCTAATGACTGGCTTTTATAAATGCCAAAACAAATTGACCATGAA
pks-RF-R	TAATGAGAGTGTGTCAATGCGACTG
Pveg-bte-LF-F	TTTATGGCGGACAAAAAGGAACTGA
P_veg_-bte-LF-F	AGTGTGATGCTGTGTAAGATAGATCGATTGACGCTCCTTTATACTCTGTA
P_veg_-F	GATCTATCTTACACAGCATCACACT
P_veg_-R	GTTTGTCCTCCTTATTAGTTAATCT
bte-F	AGATTAACTAATAAGGAGGACAAACATGGCTACAACATCTCTTGCTTCG
bte-R	ACAACAATATGGCCCGTTTGTTGAATTAAACACGAGGTTCAGCAGGGAA
Pveg-bte-DR-F	TATCCCTGCTGAACCTCGTGTTTAAATCGCATGAAAGCACATTCTCTTGA
Pveg-bte-DR-R	TCATTTGTATACATACTTTAAAAATTGAGAAAACAGCGGTATGCTGAAG
Pveg-bte-RF-F	AAATAACAGATTAAAAAAATTATAAATGTCCAAAATTATTGCAATTAAG
Pveg-bte-RF-R	TGTTTTCACCGATACCGGCAGTAAA
ackA-LF-F	TTTATGGCGGACAAAAAGGAACTGA
ackA-LF-R	CAAGAGAATGTGCTTTCATGCGATGTTTGTCCTCCTTATTAGTTAATCT
ackA-DR-F	AGATTAACTAATAAGGAGGACAAACATCGCATGAAAGCACATTCTCTTG
ackA-DR-R	TCATTTGTATACATACTTTAAAAATTGAGAAAACAGCGGTATGCTGAAT
ackA-RF-F	AAATAACAGATTAAAAAAATTATAAATGTCCAAAATTATTGCAATTAAC
ackA-RF-R	TGTTTTCACCGATACCGGCAGTAAA
amyE-up-F	AACCCGACATCCGGCGTTCTCATGG
amyE-up-R	ACAACAATATGGCCCGTTTGTTGAATCTTGACACTCCTTATTTGATTTTT
Te–F	AAAAATCAAATAAGGAGTGTCAAGATTCAACAAACGGGCCATATTGTT
Te-R	ATGTCAAAAGGAGAAGAACTTTTTA
gfp-F	ATGTCAAAAGGAGAAGAACTTTTTA
gfp-R	CGGTAAGTCCCGTCTAGCCTTGCCCTTATTTATAAAGTTCGTCCATACCG
amyE-down-R	AAGGGCAAGGCTAGACGGGACTTACCG
amyE-down-F	CACCGATGTACACGTCATCTGCAC
P_veg_ -F	TAAACATTCTCAAAGGGATTTCTAAGATCTATCTTACACAGCATCACAT
P_veg_ -R	TAAAAAGTTCTTCTCCTTTTGACAT TACATTTATTGTACAACACGAGCCC
P_43_-F	TAAACATTCTCAAAGGGATTTCTAAGATAGGTGGTATGTTTTCGCTTGAC
P_43_-R	TAAAAAGTTCTTCTCCTTTTGACATGTGTACATTCCTCTCTTACCTATAAT

Underlined letters represent complementary sequences for overlap-extension PCR

### Culture conditions and surfactin analysis

Luria–Bertani (LB) medium (1% tryptone, 0.5% yeast extract and 1% NaCl) was used for seed cultures and gene cloning. The fermentation medium contained 6% sucrose, 1% tryptone, 0.6% NaNO_3_, 0.3% KH_2_PO_4_, 1% Na_2_HPO_4_, 0.002% FeSO_4_ and 0.05% MgSO_4_. The engineered strains were precultured in LB medium and then incubated overnight at 37 °C and 200 rpm. Two milliliters of the resulting seed culture were used to inoculate a 250 ml flask with 50 ml of fermentation medium and cultured at 37 °C with shaking at 200 rpm for 36 h.

The surfactin concentration was determined using high-performance liquid chromatography (HPLC) on a U-3000 instrument (Thermo Fisher Scientific, USA) equipped with an Amethyst C18-P column (4.6 × 250 mm, 5 μm) [11]. The mobile phase was composed of 90% (v/v) methanol and 10% (v/v) water, with 0.05% trifluoroacetic acid at a flow rate of 0.8 mL/min. Authentic surfactin (98%) was purchased from Sigma Aldrich (USA). The cell growth was monitored by measuring the optical density at 600 nm (OD_600_).

### Promoter relative strength measurements

The relative strength of promoters was measured by whole cell fluorescence of strains 168 (P_veg_-GFP) and 168 (P_43_-GFP). The coding sequence of green fluorescent protein GFP fused with P_veg_ promoter or the P_43_ promoter, was inserted at the *amyE* locus of the genome of *B. subtilis 168* using the primers shown in Table 2. The expression of GFP from the different promoters was monitored by measuring whole-cell fluorescence using a Spectra Max M3 multimode microplate reader (Shanghai Huanxi Medical Equipment Co., Ltd, China). After culturing for 12 h, 1 ml of fermentation broth was centrifuged at 10,956×*g* for 10 min, the supernatant was discarded, and the cells were washed 3 times. The washed cells were re-suspended in deionized water to the same optical density (OD_600_). The excitation wavelength and emission wavelengths were 485 and 525 nm respectively. *B. subtilis* 168 without the chromosomal *gfp* expression cassette was used as the negative control. Standard deviations are based on a minimum of three statistically independent experiments.

### Isolation, purification and isoform analysis of surfactin

Surfactin was extracted and purified using the acid precipitation method [26]. After fermentation, the cells were removed by centrifugation at 10,956×*g* for 10 min. Then, 6 mol/L HCl was added to the supernatant to achieve a pH of 2.0 for acid precipitation, and allowed to settle at 4 °C overnight. The acid precipitate was collected by centrifugation at 10956×*g* for 10 min. The final pH was adjusted to 7.0 with 5 mol/L NaOH, and the neutralized precipitate was lyophilized. The dried surfactin components were further extracted with methanol and dried on a rotary evaporator under vacuum.

Surfactin components were analyzed by reverse-phase UPLC–MS (UPLC, Agilent, 1290) coupled with a single quadrupole MS (Q-TOF, Agilent, 6550) on an extend C18 column (2.1 × 50 mm 1.7 µm; Agilent) using a method based on the acetonitrile/water (acidified with 0.1% formic acid) gradient that allowed the simultaneous detection of all three lipopeptide families. Elution was started at 10% acetonitrile at a flow rate of 0.50 mL/min. After 7 min, the percentage of acetonitrile was increased to 95% and held until 5 min. Then, the column was re-equilibrated with 10% acetonitrile for 1 min. The compounds produced by BSFX024 and BSFX025 were compared. Ionization and source conditions were set as follows: source temperature, 150 °C; desolvation temperature, 350 °C; nitrogen flow, 15 L/min; voltage, 4000 V.

### Fatty acid side-chain analysis

The fatty acid side-chains were analyzed according to the method reported by Zhao et al. [27]. Briefly, 10 mg of the purified surfactin were hydrolyzed with 6 mol/L HCl in an ampoule at 90 °C for 20 h, and the solvent was subsequently removed by blowing air at 60 °C. Then, 500 μL acetonitrile-BSTFA (*N*,*O*-bis(trimethylsilyl)-trifluoroacetamide) (3: 2 by vol) was added to the sample, and reacted at 60 °C for 20 min. Subsequently, the samples were analyzed by GC–MS. GC–MS analysis was performed on a 6890–5975 C gas chromatography–mass spectrometry instrument (GC–MS; Agilent, USA) equipped with an HP-5 MS capillary silica column (60 m × 0.25 mm × 0.25 μm, Agilent, USA). The ions were obtained by electron ionization EI at 70 eV using a source temperature of 230 °C. The column oven temperature was kept initially at 60 °C for 3 min, increased to 250 °C at a rate of 10 °C/min, and held for 5 min. The other conditions were as follows: helium carrier gas, 99.999%; flow rate, 1.0 mL/min; injector temperature, 250 °C; injector volume, 1 μL; split ratio, 20:1.

### Critical micelle concentration (CMC) of the biosurfactant

As the concentration increases, the decreasing rate of surface tension will suddenly change at CMC [26]. The surfactin samples obtained from recombinant strains BSFX024 and BSFX025 were dissolved in distilled water at different concentrations (0–100 mg/L). Then, the surface tension was measured using the platinum plate method on an automated tensiometer (BZY-3B; Shanghai Automation Instrumentation Sales Center, China) at 25 °C. The CMC was determined based on the inflection point of surface tension versus concentration.

### Measurement of emulsification activity

For the measurement of emulsification activity, 2 mL of a solution containing 200 mg/L surfactin obtained from recombinant strain BSFX024 or BSFX025 and 2 mL of different hydrocarbons (dodecane, tetradecane, hexadecane, octadecane, p-xylene and liquid paraffin) were mixed in cylindrical glass vials, respectively. The mixtures were vortexed at maximum speed (QT-2; Qite Corp., Shanghai, China), and then incubated at 25 °C for 24 h. The emulsification activity was calculated using the emulsification index (EI_24_) formula [4]:

$$\text{Emulsification Index (EI24)}=\frac{\text{Height of the emulsion layer}}{\text{Height of the organic phase layer}} \times 100\%$$

### Oil washing efficiency

The standard oil sand for measuring the oil-washing efficiency was prepared according to a reported method [11, 28]. Briefly, 170 g of quartz sand, 4 g of artificial crude oil (Shengli Oilfield, China) and 10 ml of petroleum ether were mixed. The mixtures were heated at 80 ℃ for 1 h to remove the petroleum ether and then aged at 60 ℃ for 7 days. Subsequently, 2 g of the aged oil sand was placed into a flask with 20 ml of different surfactin preparations with different concentrations (0.05 g/L, 0.1 g/L, 0.15 g/L and 0.2 g/L). Then, the flasks were shaken at 90 rpm and 70 ℃ for 12 h. The sand containing residual oil was dried at 80 ℃ for 12 h and the removed oil remaining in the solvent fraction was further extracted with petroleum ether. The absorbance at 225 nm was measured (722S UV–vis spectrometer; Shanghai Precision Instrument Co., China) to calculate the oil washing efficiency of the surfactin preparations using the formula:

$$\text{Oil washing efficiency} = \frac{\text{The removed oil in solution}}{\text{Total oil in the sand}}{\times 100\%}$$

### Contact angle measurements

Wettability was determined by measuring the contact angle of the aqueous surfactant solution on an oil film. The oil film was prepared by painting a thin layer of crude oil onto a glass slide, which was then aged at 80 ℃ for 7 days. The contact angle of the surfactant solutions with different concentrations (0.05 g/L, 0.1 g/L, 0.15 g/L and 0.2 g/L) on the oil film was measured using a drop-shape analyzer (DropMeter A100P; Ningbo Ouyi Testing Instrument Co., Ltd, China).

## Results

### Influence of enhancing precursor supply on the production of surfactin

**Fig. 1:**
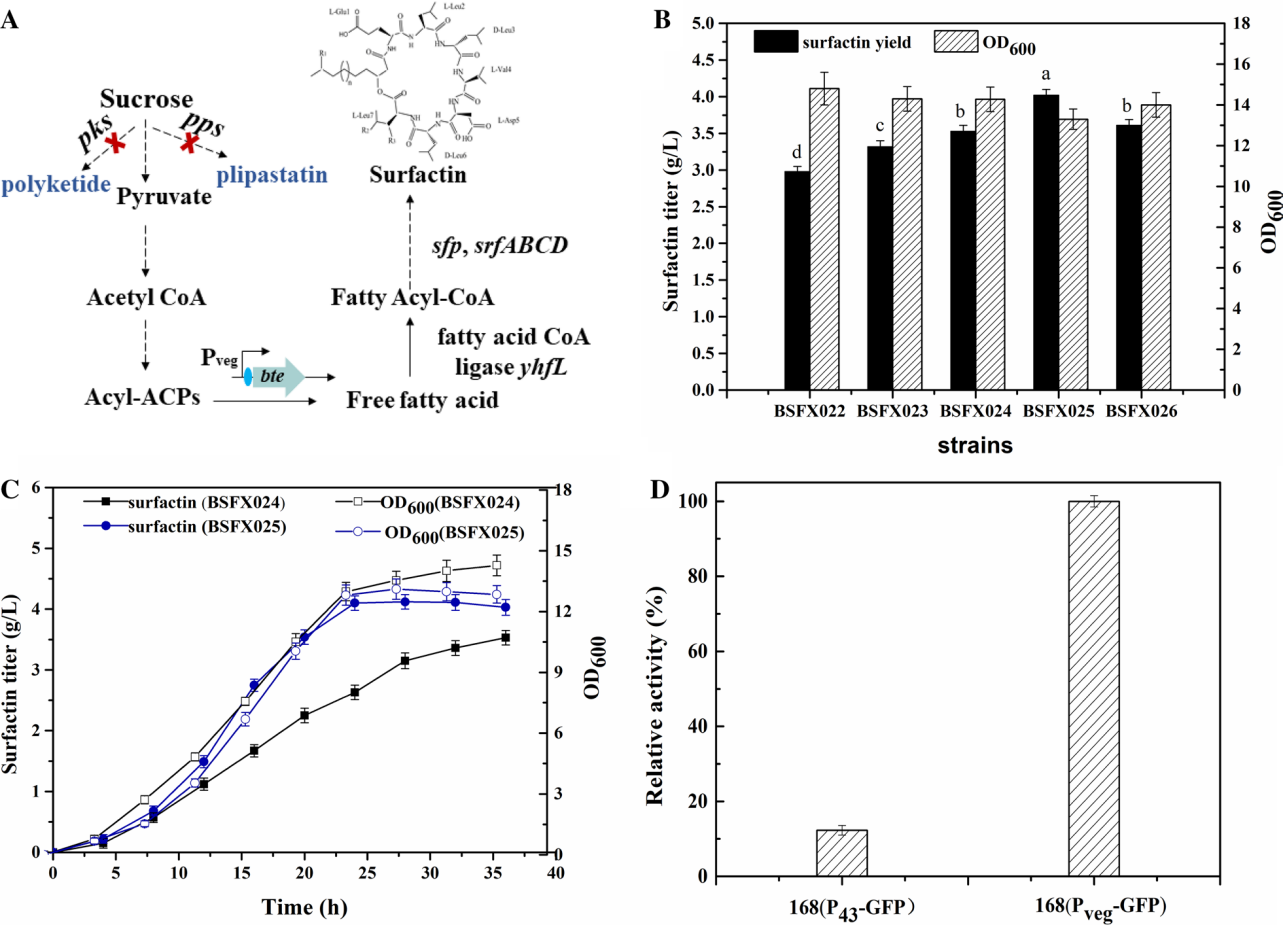
Effects of metabolic modifications on the fermentation of surfactin. **a** The diverse metabolic modifications made for improving the titre of surfactin. **b** The surfactin production and OD_600_ of various recombinant strains after 36 h of incubation. **c** Time profiles of cell growth and surfactin production of strains BSFX024 and BSFX025. **d** The strength of the P_veg_ and P_43_ promoters according to the GFP fluorescence measured using a microplate reader. Error bars indicate standard deviation for triplicate experiments. The superscript letters in the **b** were the results of Dunnett-t test (a = 0.05)

Expression of BTE in *Escherichia coli* was reported to promote the conversion of long-chain acyl ACP to C_12_ and C_14_ free fatty acids [29]. Fatty acids are important precursors for the synthesis of surfactin and the core of the precursor supply module in the synthesis pathway of surfactin [30]. Here, to reduce competition for direct precursors, the nonribosomal peptide plipastatin synthetase gene *pps* and polyketide synthase gene *pks* were knocked out, as shown in Fig. 1a. The obtained mutants were named BSFX023 (Δ*pps*) and BSFX024 (Δ*pps*, Δ*pks*). Subsequently, the *bte* coding sequence under the control of the P_veg_ promoter was integrated into the *ackA* locus (acetate kinase gene) to obtain the mutant BSFX025 (Δ*pps*, Δ*pks*, *bte*). In order to further demonstrate the effect of *bte* on the synthesis of surfactin, the mutant BSFX026 (Δ*pps*, Δ*pks*, Δ*ackA)* was constructed with the inactivation of acetate kinase gene *ackA*. As shown in Fig. 1b, the surfactin production of control strain BSFX022 was 2.98 ± 0.075 g/L. The obtained mutants BSFX024 and BSFX025 produced 3.32 ± 0.080 g/L and 3.51 ± 0.083 g/L of surfactin, respectively, which increased by 11.40% and 18.55% compared to BSFX022. The surfactin titer of BSFX025 reached 4.02 ± 0.085 g/L, representing a 34% improvement compared to BSFX022.The surfactin production of BSFX026 was 3.60 ± 0.081 g/L, almost the same as that of BSFX024. The statistical analysis results obtained by software SPSS 22.0 shown that the surfactin accumulation of BSFX026 and BSFX024 were not significant, which indicated that the surfactin overproduction of BSFX025 was mainly contributed by *bte* gene overexpression. To further analyze the influence of BTE, the surfactin accumulation and strain growth were compared between BSFX024 and BSFX025, as depicted in Fig. 1c. Although the expression of BTE had a weak inhibitory effect on the growth of the strain, the production rate of surfactin increased from 0.112 to 0.177 g/L/h at 24 h. These results indicated higher availability of free fatty acids is very beneficial to the synthesis of surfactin through the thioesterase activity of BTE.

In our previous research, integrating the *bte* coding sequence and *yhfL* under the control of the strong constitutive promoter P_43_ into the genome together with knocking out of *fadE* significantly improved the production of surfactin by 2.39 times [21]. Here, the *bte* coding sequence was placed under the control of the strong promoter P_veg_, in which the − 35 and − 10 regions were optimized for high transcriptional efficiency [25]. The transcription intensity of P_veg_ and P_43_ was identified by measuring the GFP activity. Figure 1d illustrated the strength of the P_veg_ and P_43_ promoters according to the GFP fluorescence measured using a microplate reader. P_veg_ was more than eightfold stronger than the P_43_ promoter. In addition, this manipulate also increased the copy of the *bte* gene on the genome of *B. subtilis* 168. This may be the reason why the output of surfactin was further improved in the corresponding strain.

### HPLC–MS analysis of the isomeric composition of surfactin

**Fig. 2:**
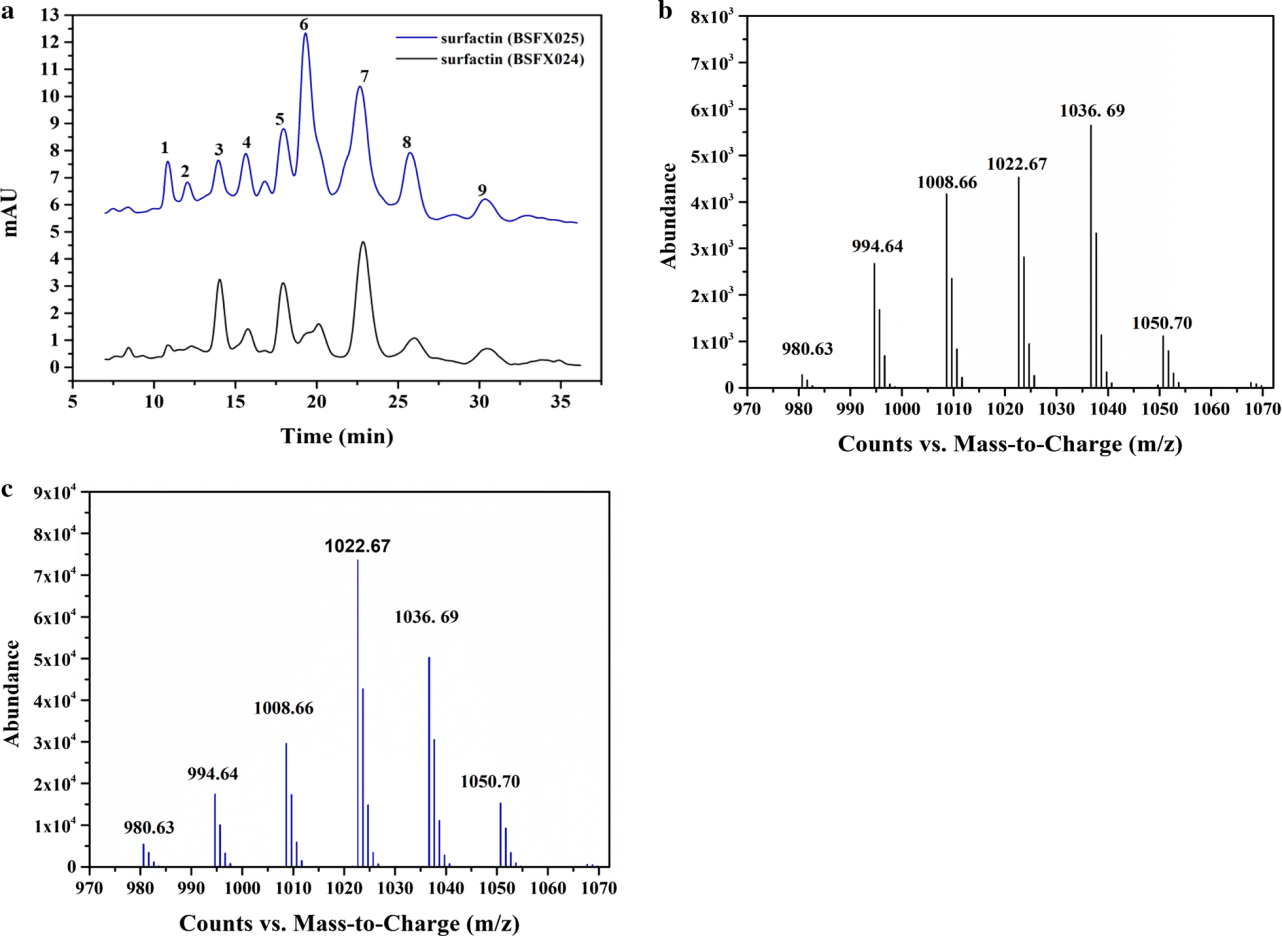
HPLC–MS analysis of the isomeric composition of surfactin. **a** The HPLC profile of surfactin produced by the engineered strains BSFX024 and BSFX025. **b** The ESI–MS spectrum of surfactin produced by BSFX024. **c** The ESI–MS spectrum of surfactin produced by BSFX025

HPLC analysis indicated that the overexpression of BTE also caused changes in the isomeric composition of surfactin. As shown in Fig. 2, a total of nine components were detected and there were obvious differences in the surfactin peaks of BSFX025 and BSFX024, especially the peaks at positions 1, 2, and 6. To further confirm the isomeric components of surfactin at each position, ESI–MS was performed. The molecular weight information of each component was obtained by mass spectrometry analysis (Fig. 2b, c). According to the results of mass spectrometry, we deduced the likely structure represented by each peak, as summarized in Table 3. The study of the peptide structure showed that Glu, Leu, Asp and Leu at positions 1, 3, 5 and 6 are usually conserved, and the amino acids at positions 2, 4 and 7 can be replaced by Leu, Val or Ile [31]. Due to the specificity of the C-domain of non-ribosomal peptide synthase (NRPS) for substrate recognition, the fatty acid chain length of surfactin is usually 13–15 [32].

**Table 3:** Likely identity and structural formula deduced for each peak

Number	m/z	Molecular formula	Speculated structure
1	980.63	([C_49_H_85_N_7_O_13_] + H)^+^	C_12_[Leu/Ile^2^, Val^4^, Val^7^] or C_13_[Val^2^, Val^4^, Val^7^]
2	994.64	([C_50_H_87_N_7_O_13_] + H)^+^	C_13_[Leu/Ile^2^, Val^4^, Val^7^] or C_12_[Leu/Ile^2^, Val^4^, Leu/Ile^7^]
3	1008.66	([C_51_H_89_N_7_O_13_] + H)^+^	C_13_[Leu/Ile ^2^, Val^4^, Leu/Ile ^7^]
4	994.64	([C_50_H_87_N_7_O_13_] + H)^+^	C_13_[Leu/Ile ^2^, Val^4^, Val^7^]
5	1022.67	([C_52_H_91_N_7_O_13_] + H)^+^	C_14_[Leu/Ile ^2^, Val^4^, Val^7^]
6	1022.67	([C_52_H_91_N_7_O_13_] + H)^+^	C_14_[Leu/Ile ^2^, Val^4^, Val^7^]
7	1036.69	([C_53_H_93_N_7_O_13_] + H)^+^	C_15_[Leu/Ile ^2^, Val^4^, Leu/Ile ^7^]
8	1036.69	([C_53_H_93_N_7_O_13_] + H)^+^	C_15_[Leu/Ile ^2^, Val^4^, Leu/Ile ^7^]
9	1050.70	([C_54_H_95_N_7_O_13_] + H)^+^	C_15_[Leu/Ile ^2^, Leu/Ile ^4^, Leu/Ile ^7^]

According to the structural characteristics of surfactin and the m/z of each component, the likely structure represented by each peak was deduced as summarized in Table 3. The molecular mass of peak 1 was 979, with the major [M + H]^+^ peak at m/z 980.63 the same as the reported of Fei et al. [33]. Therefore, its structure may be C_12_[Leu/Ile^2^, Val^4^, Val^7^] or C_13_[Val^2^, Val^4^, Val^7^]. At the same time, we speculated that the structure of component 2 may be C_12_[Leu/Ile2, Val4, Leu/Ile7] or C_13_[Leu/Ile2, Val4, Val7]. Peaks with molecular masses of 1008, 1022 and 1036 have been proved to contain C_13_-surfactin, C_14_-surfactin and C_15_-surfactin in our previous work [34]. It can be seen that the overexpression of BTE obviously enhanced the production of C_14_-surfactin and may even induce the production of C_12_-surfactin. However, to further confirm the detailed isoform of the fatty acid side-chain, GC–MS analysis was needed.

### Isoforms of the fatty acid side-chain

For fatty acid analysis, after acidic hydrolysis and trimethylsilylation of the surfactin sample obtained from strain BSFX024 and BSFX025, the composition was evaluated using a gas chromatography mass spectrometer (GC–MS). The BSTFA (N,O-bis(trimethylsilyl)-trifluoroacetamide) was reacted with the fatty acid moiety of surfactin, which consisted of β-hydroxyl fatty acid with different carbon atoms, replacing active hydrogens with a -Si(CH_3_)_3_ (trimethylsilyl) group[27]. The characteristic m/z of 233 in each mass spectrogram was the characteristic fragment ion of the β-hydroxyl fatty acid, which suggested that one hydrogen and one methyl on the –[CH(OH)CH_2_COOCH_3_]^+^ was replaced by –Si(CH_3_)_3_ groups, resulting in the structure of -[CHO(Si(CH_3_)_3_)CH_2_COOSi(CH_3_)_3_]^+^. Isoforms of the branched chains of the fatty acid were determined by referring to the literature and comparing the value of I_43_/I_57_ [35].

**Fig. 3:**
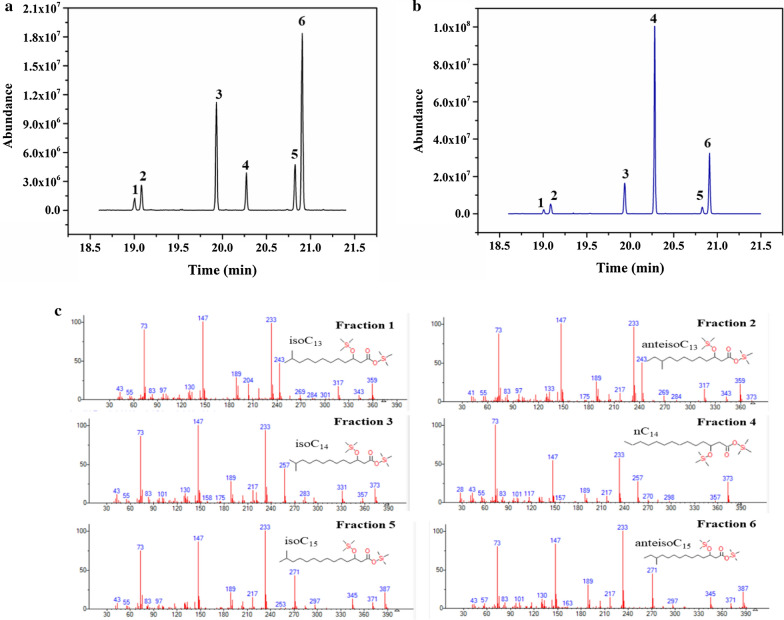
GC–MS analysis of β-hydroxy fatty acids in the surfactin produced by strains BSFX024 and BSFX025 after trimethylsilylation. **a** Extracted ion (m/z = 233) chromatograms of the surfactin from strain BSFX024. **b** Extracted ion (m/z = 233) chromatograms of the surfactin produced by strain BSFX025. **c** Mass spectrograms of the β-hydroxy fatty acid fraction after trimethylsilylation

The extracted ion chromatograms (m/z = 233) of the surfactin sample obtained from strains BSFX024 and BSFX025 are displayed in Fig. 3a, b. Six fractions were observed in the total ion chromatogram of the surfactin samples and the proportions of these fractions showed obvious differences in the two samples, especially fraction 4. These fractions were identified as *iso*C_13_, *anteiso*C_13,_ *iso*C_14,_ *n*C_14,_ *iso*C_15_ and *anteiso*C_15,_ respectively, on the basis of the MS Agilent NIST 05 Chemical Structure Library (Fig. 3c). The proportion of each fatty acid component in the two surfactin samples was further summarized in Table 4, which clearly showed that the proportion of *n*C_14_-surfactin in the extract of strain BSFX025 was 6.4 times higher than that of BSFX024.

**Table 4:** Likely identity and structural formula of the fatty acid moiety deduced for each peak

Fraction	Fatty acid in surfactin	Relative proportion (%)	
		BSFX024	BSFX025
1	*iso*C_13_	3.24 ± 0.4	1.67 ± 0.2
2	*anteiso*C_13_	6.55 ± 0.44	4.42 ± 0.43
3	*iso*C_14_	25.77 ± 0.6	13.08 ± 0.52
4	*n*C_14_	8.76 ± 0.45	56.13 ± 0.7
5	*iso*C_15_	11.61 ± 0.52	2.94 ± 0.2
6	*anteiso*C_15_	44.06 ± 0.67	21.77 ± 0.56

^a^Results represent the average of three independent experiments ± standard deviation

### Changes in the surfactant properties of ***n***C_14_-rich surfactin

Because the FabH enzyme of *B. subtilis* responsible for initiating the straight- and branched-chain fatty acid synthesis cycle by condensing acetyl-CoA, isobutyryl-CoA, isovalerylCoA or α-methylbutyryl-CoA with malonyl-ACP shows higher activity with branched-chain fatty acid synthesis precursors, the branched-chain configuration (*iso*C_13,_ *anteiso*C_13,_ *iso*C_14,_ *iso*C_15_ and *anteiso*C_15_) accounts for the highest proportion of the β-hydroxy fatty acid chains of surfactin [35]. In this study, the straight-chain fatty acid configuration became the main component in the β-hydroxy fatty acid side-chain of surfactin produced by strain BSFX025, which means that the properties and functional activity of surfactin may change accordingly. In order to further understand the influence of fatty acid chain length and configuration on surfactin activity, the critical micelle concentration (CMC), emulsification activity, oil-washing efficiency and wettability of two surfactin samples were compared.

#### Comparison of the critical micelle concentration

**Fig. 4:**
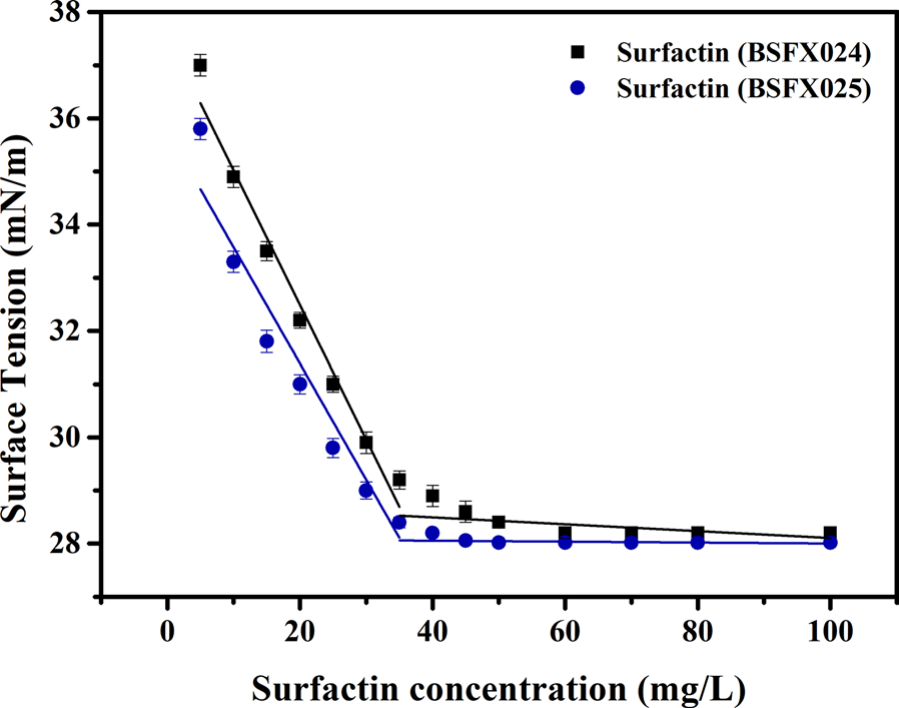
Determination of the CMCs of surfactin produced by strains BSFX024 and BSFX025. The error bars represent standard deviation values of three independent experiments (n = 3)

CMC is a crucial parameter in the assessment of biosurfactants. Figure 4 exhibits the CMC of the two surfactin samples was assessed by measuring the surface tension of solutions with different surfactin concentrations (0–100 mg/L). It was observed that the CMC of surfactin obtained from BSFX024 was about 38 mg/L, while the CMC of the surfactin obtained from BSFX025 was about 36 mg/L. Although the CMC values of the two samples were similar, the surface tension of surfactin from BSFX025 was relatively low in the low concentration range.

#### Oil-washing efficiency of the surfactin produced by the two strains

**Fig. 5:**
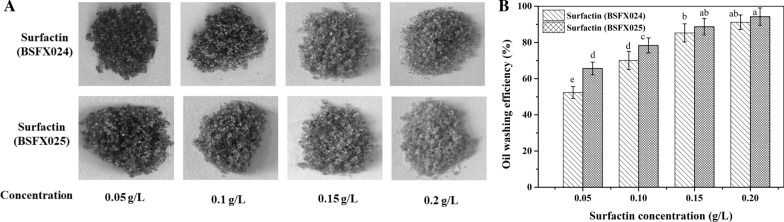
The oil washing efficiency of the two surfactin preparations at different concentrations. **a** Photographs of dried oil sand after washing. **b** The oil washing efficiency corresponding to the samples shown in A. Error bars indicate standard deviation for triplicate experiments. The superscript letters in the Fig. 5b were the results of Dunnett-t test (a = 0.05)

The oil-washing efficiency can indirectly reflect the potential of surfactin for applications in MEOR. The performance of the surfactin produced by strains BSFX024 and BSFX025 in oil-sand washing at different concentrations is presented in Fig. 5. Figure 5a shows photographs of dried oil sand after washing with different concentrations of surfactin. The definite oil-washing efficiency value of the different surfactin solutions was further quantified as shown in Fig. 5b. Through statistical analysis by software SPSS 22.0, it can be seen that the differences of oil-washing efficiency between two samples under relatively high concentrations (above 0.15 g/L) were not significant, while the differences became more significant as the concentration was lower than 0.1 g/L. The oil-washing efficiency of the two surfactin preparations was above 85% in both cases, and the color of the oil sands after washing were similar at concentrations of 0.2 and 0.15 g/L. However, at concentrations of 0.1 and 0.05 g/L, the color of the oil sands washed with the surfactin from strain BSFX025 was slightly lighter than that of the oil sand washed with the surfactin form strain BSFX024. Accordingly, the oil-washing efficiency was also relatively higher by 9.2% and 14.5%. Because the oil-washing efficiency in reservoirs is influenced by two successive processes, stripping and emulsification of crude oil, it is necessary to compare the emulsification and wettability alteration capability of the two surfactin preparations.

#### Emulsification activity of surfactin preparations produced by the two strains

**Fig. 6:**
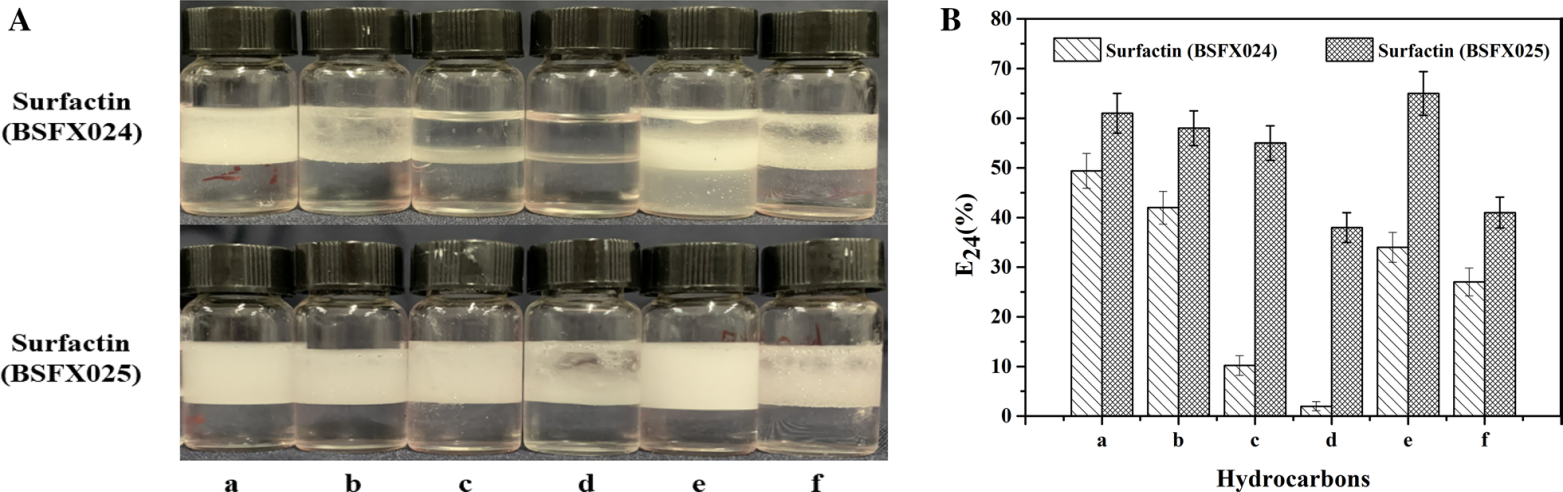
Emulsification of different hydrocarbons by the two surfactin preparations at 200 mg/L. **a** Photographs of the emulsion after 24 h. **b** The emulsification index after 24 h (E_24_) for different substrates. a. dodecane. b. tetradecane. c. hexadecane. d. octadecane. e. p-xylene. f. liquid paraffin. Error bars indicate standard deviation for triplicate experiments

The amphipathic structure of surfactin gives it emulsifying properties. This feature can be used to reduce the viscosity of heavy oil, which is rich in asphaltenes and gums, and its high viscosity and poor fluidity cause great difficulties in oil exploitation [36]. Surfactin can emulsify strongly hydrophobic substrates such as pentadecane, diesel, hexane and kerosene [37–40]. A comparison of the emulsification activity of the two surfactin preparations at 200 mg/L is shown in Fig. 6. Figure 6a manifests the emulsification of different alkanes (dodecane, tetradecane, hexadecane, octadecane, p-xylene and liquid paraffin) by two the surfactin preparations, whereby the surfactin from strian BSFX025 showed obvious advantages in the emulsification of long-chain alkanes. The O/W (oil-in-water) emulsion layers of tetradecane, hexadecane and *p*-xylene with the surfactin from strain BSFX025 were thick and tight, whereas the emulsified layers with the surfactin from strain BSFX024 were loose or almost invisible. Furthermore, Fig. 6b shows the emulsification index at 24 h (E_24_) of the two surfactin preparations with different substrates, from which it can be more intuitively seen that the surfactin from strain BSFX025 had better emulsification activity.

#### Analysis of changes in surface wettability by surfactin produced by the two strains

**Fig. 7:**
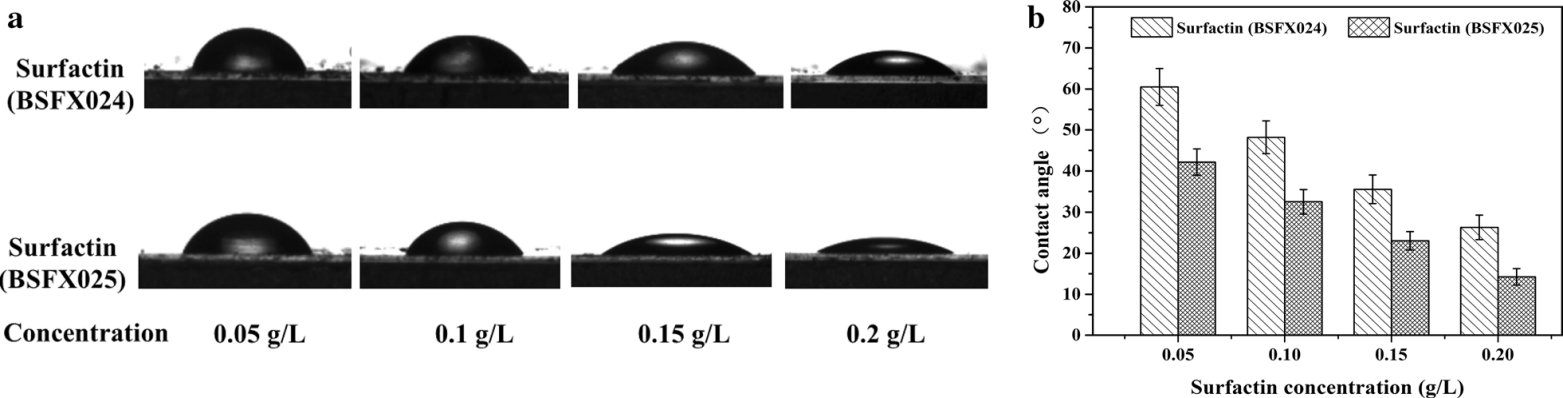
Change of wettability by the two surfactin preparations. **a** The drop shape in the gas–liquid-solid three-phase contact angle experiment. **b** The contact angles of the corresponding samples in A. Error bars indicate standard deviation for triplicate experiments

The ability of the two surfactin preparations to alter the wettability of solid surface was analyzed by measuring the contact angle of surfactin-containing water drops on an oil film. The shapes of the surfactin solution drops on the oil-wet solid surface and the contact angle value of the corresponding samples were shown as Fig. 7a, b. As the surfactin concentration increased, the adsorption of surfactin solution on the oil film surface gradually became tighter, and the contact angle decreased accordingly. Although the two surfactin preparations showed the same trend, there still were distinct changes. The contact angle of the solution containing surfactin from strain BSFX025 on the silicate solid surface decreased from 45.7 to 17.8° for an increase of surfactant concentration from 0.05 to 0. 2 g/L, while the contact angle of the solution containing surfactin from strain BSFX024 was in the range of 60.2 to 26.6°. The obtained results revealed that the surfactin from strain BSFX025 was more effective in altering the wettability of the oil-saturated silicate solid surface towards a more hydrophilic state.

## Discussion

In this work, the overexpression of the plant thioesterase BTE changed the proportion of surfactin isoforms, resulting in surfactin with the *n*C_14_ isoform as the predominant component. As reported, two kinds of thioesterases are useful in fatty acid production, the TesA thioesterase from *E. coli* and medium chain thioesterases from plants*.* Wu et al. [30] overexpressed the TesA thioesterase from *E. coli* with a strong preference for long-chain acyl-ACP substrates and deleted the first gene of the fatty acid degradation pathway in the engineered *B. subtilis* 168, which increased the output of surfactin by nearly 55% [41]. Regretfully, they did not report if there were any changes in the isomeric composition of surfactin. In fact, the proportion of *n*C_14_ component is very small among the reported surfactin production strains (Table 5). For example, among the β-hydroxyl fatty acids of surfactin produced by the natural strain *B. subtilis* HSO121, the *n*C_14_ component only accounts for 8.95% of the total [27], which is consistent with the results of our control strain BSFX024. The proportion of these components can be altered to a certain extent by changing the medium composition, adding branched chain amino acids and specific genetic modifications. However, most of these changes are reflected in the *iso-* and *anteiso-*components, while an improvement of the *n-*component is rarely seen. The only approach to specifically overproduce surfactin with a C_14_ FA chain was reported by Dhali et al. [17], who increased the proportion of the surfactin C_14_ isoform 2.5 times through genetic engineering of the branched-chain amino acid degradation pathway. To our best knowledge, this is the first study to show that the overexpression of thioesterase BTE can increased the proportion of *n*C_14_-surfactin by 6.4 times.

**Table 5:** Reported proportions of different isoforms in surfactin samples produced by different strains

Strains	Relative proportion (%)						Reason for proportion change	References
	**C**_**13**_		**C**_**14**_		**C**_**15**_			
	*iso*C_13_	*anteiso*C_13_	*iso*C_14_	*n*C_14_	*iso*C_15_	*anteiso*C_15_		
*B. subtilis* BS-37	13.3		35.2		51.6		Change of medium composition	[11]
	15.5		10.3		74.2			
*B. velezensis* BS‐37	14.4		16.7	14.5	51.8		Addition of the branched chain amino acid L-Leu	[42]
	28.3		4.2	10.1	57.4			
*B. subtilis* BSB1	39.7		21.2		28		Knockout of *codY* and *lpdV*	[17]
	7.7		52.7		25			
*B. subtilis* THY-7	5		51.8	4.1	39.1		Replacement of P_srfA_ with P_g3_	[43]
	16.8		32.6	19.1	31.5			
*B. subtilis* HSO121	4.89	6.27	23.05	8.95	17.69	38.69	Natural strain	[27]
*B. subtilis* BSFX025	1.67	4.42	13.08	56.13	2.94	21.77	Overexpression of *bte*	This work

The surface activity was enhanced due to the increase in the percentage of *n*C_14_-surfactin. In addition, it was observed that the product with more *n*C_14_-surfactin was more effective in washing oil sand, emulsifying hydrocarbons and altering the wettability of oil-wet solid. Youssef et al. [14] showed that the optimal hydrophilic–lipophilic balance required for the highest surface activity is exhibited by C_14_-surfactin. Similarly, Bacon et al. [44] proved that C_14_-surfactin possesses higher surface activity. This is consistent with our research results, indicating that C_14_-surfactin has the best surface activity among all the surfactin components reported so far. Similarly, Yakimov et al. [45] found that an increase in the percentage of branched-chain fatty acids in lichenysin A decreased the surface activity, whereas an increase in the percentage of straight-chain 3-hydroxytetradecanoate (*n*-3OH-C_14_) enhanced the surface activity. Liu et al. [11] reported that a higher C_15_-surfactin content leads to better oil-washing efficiency at low concentrations. We found that the product with more *n*C_14_-surfactin also had a better performance in oil-washing. This phenomenon signifies that the isomerism of the fatty acid side-chain may have a greater influence on the activity of surfactin than the length. The emulsification activity also depends on the structure of the biosurfactant. According to Tao et al. [46], the hydrophilic group of surfactin stretches towards the water phase and the lipophilic group inserts itself into the oil phase, forming a dense film on the interface. The hydrophobic fatty acid chain of the surfactin produced by strain BSFX025 has a higher percentage of straight-chain 3-hydroxytetradecanoate. As a consequence, the emulsifying activity also changed significantly. The emulsification capacity observed for the surfactin produced by strain BSFX025 suggests that it can potentially be used in the oil industry for cleaning the sludge in storage tanks, oil mobilization and MEOR.

Wettability alteration towards a strongly hydrophilic state is a favorable modification for obtaining higher oil recovery rates, because capillary forces will change from negative towards positive values due to this wettability alteration [47]. As a result, the changed surface activity is more conducive for water passing through porous rock, thereby further enhancing the displacement of trapped oil. The mechanisms of wettability alteration by sodium dodecyl benzene sulfonate (SDBS) and cationic dodecyltrimethyl-ammonium bromide (C12TAB) indicates that the extent of wettability alteration is dictated by the structure of the applied surfactant. SDBS cannot alter the wettability towards a strongly hydrophilic state because of the larger numbers of hydrophobic side chain groups [48]. By contrast, C12TAB can induce better water-wettability of the surface due to the formation of ion pairs between the cationic heads of C12TAB and the acidic components of crude oil absorbed onto the rock surface [49]. Biosurfactants have been proved to alter the wettability of surfaces to the same extent as chemical surfactants, whereby the alteration of wettability is also related to the isomeric structure [50]. Here, the change in the hydrophobic fatty acid side-chain of surfactin caused the difference in wettability alteration of the two surfactin preparations. The increase in the percentage of straight-chain 3-hydroxytetradecanoate led to stronger surface changes and better water-wettability. Further experiments should be carried out for improving the production of *n*C_14_-surfactin and scaling up its application.

## Data Availability

The dataset supporting the conclusions of this article is included within the article (and its additional file).

## Abbreviations

MEOR: Microbial enhanced oil recovery
ACP: Acyl-carrier protein
CMC: Critical micelle concentration
SDBS: Sodium dodecyl benzene sulfonate
C12TAB: Cationic dodecyltrimethyl-ammonium bromide (C12TAB
